# Autistic women’s diagnostic experiences: Interactions with identity and impacts on well-being

**DOI:** 10.1177/17455057221137477

**Published:** 2022-11-15

**Authors:** Miriam Harmens, Felicity Sedgewick, Hannah Hobson

**Affiliations:** 1 Department of Psychology, University of York, York, UK; 2 School of Education, University of Bristol, Bristol, UK

**Keywords:** autism, diagnosis, identity, well-being, women

## Abstract

**Objective:**

There has been suggestion that current diagnostic instruments are not sufficient for detecting and diagnosing autism in women, and research suggests that a lack of diagnosis could negatively impact autistic women’s well-being and identity. This study aimed to explore the well-being and identity of autistic women at three points of their diagnostic journey: self-identifying or awaiting assessment, currently undergoing assessment or recently diagnosed, and more than a year post-diagnosis.

**Methods:**

Mixed-methods were used to explore this with 96 women who identified as autistic and within one of these three groups. Participants completed an online questionnaire, and a sub-sample of 24 of these women participated in a semi-structured interview.

**Results:**

Well-being was found to differ significantly across groups in three domains: satisfaction with health, psychological health, and environmental health. *Validation* was found to be a central issue for all autistic women, which impacted their diagnosis, identity, and well-being. The subthemes of *don’t forget I’m autistic; what now?; having to be the professional*; and *no one saw me* were also identified.

**Conclusion:**

These results suggest that autistic women’s well-being and identity differ in relation to their position on the diagnostic journey in a non-linear manner. We suggest that training on the presentation of autism in women for primary and secondary healthcare professionals, along with improved diagnostic and support pathways for autistic adult women could go some way to support well-being.

## Introduction

Autism is characterized by social communication and interaction difficulties as well as restricted and repetitive behavioural patterns and sensory sensitivities.^1^ Gender differences in autism diagnosis in adulthood have been found, with the proportion of women seeking a diagnosis increasing with age, and gender ratios of prevalence ranging from 1 woman for every 1–3 men.^2^ This suggests that the prevalence estimates of autistic women may be lower than reality due to many now being diagnosed later in adulthood. The aim of this study is to explore the interaction between diagnosis and the diagnostic process, identity, and well-being for adult autistic women.

Some have argued that there could be a ‘female autism phenotype’ which is not picked up by current diagnostic instruments, contributing to females being misdiagnosed or missed altogether, leading to the diagnostic differences seen between females and males with autism.^3^ The phenotype suggested includes females camouflaging their difficulties, leading to them not being recognized; their restricted and repetitive interests being seen as more ‘typical’ for their age and therefore not being seen as autistic symptoms; and a higher co-occurrence of internalizing disorders.^3,4^ Indeed, examining the role of gender in diagnostic instruments, studies have found that most of the diagnostic tools used currently were developed based largely on the observation of boys, and that many diagnostic tools, including the Autism Diagnostic Observation Schedule (ADOS) and Autism Diagnostic Interview-Revised (ADI-R), are less sensitive to women and girls.^5^ Others have found six traits and behaviours which consistently show gender differences, and which are a barrier to young autistic women gaining an autism diagnosis: behavioural problems, social and communication abilities, additional diagnoses/misdiagnosis, relationships, language, and repetitive and restrictive behaviours and interests.^6^ Driver and Chester^5^ support this, showing that many autistic women are first mis/diagnosed with mental health conditions, overshadowing an underlying autism diagnosis and stopping further investigation.

Understanding the process of diagnosis from autistic women’s perspective is crucial. While diagnosis might open up additional support and understanding, there may be factors that influence whether the process has a positive effect. The stereotype of autism as a ‘male’ disorder, even within healthcare, can be a barrier to women gaining an autism diagnosis; Driver and Chester^5^ found professionals involved in primary care and in diagnosis to be lacking in knowledge and training, while Lockwood Estrin et al.^6^ found parental concerns, others’ perceptions, a lack of information and resources, clinical bias, and compensatory behaviours on the women’s part to be perceived barriers to diagnosis by autistic women. Barriers to accessing healthcare may be one of the greatest difficulties within the process of diagnosis for autistic individuals, with 80% of autistic adults reporting difficulties around visiting their general practitioner.^7^ This was associated with increased adverse health outcomes for autistic adults, and it may be that these challenges mean diagnosis itself is not accessible to all autistic individuals. In addition, autism stereotypes continue post-diagnosis, impacting autistic women’s access to support due to the support available being tailored to men or those with a co-occurring intellectual disability.^8^

Timely diagnosis can have a significant impact on autistic women and girls’ well-being, with studies showing improved well-being post-diagnosis, and those who are undiagnosed having worse outcomes. With autistic women being more likely to be diagnosed later in life than men, some may argue that they have made it this far and therefore do not need a diagnosis. However, the known negative impact of being undiagnosed on well-being means that even if diagnosis comes later in life, it is still valuable in facilitating an improved self-understanding and increased access to support.^9^ Without a diagnosis, autistic women have been shown to have increased vulnerability due to social difficulties, which can lead to exploitation.^10^ Therefore, timely diagnosis is crucial in improving autistic women’s well-being and safety generally. However, the extent to which diagnosis improves well-being has been suggested to rely on the level of acceptance both by oneself and others of autistic women.^11^ This study found that autistic women can be exhausted by the diagnostic process, which is exacerbated by the stereotypes they face and the lack of acceptance and understanding of themselves and from others. Therefore, timely diagnosis and an increase in knowledge and understanding around autism, especially autistic women’s presentation, may be key in improving autistic women’s well-being.

Diagnosis (or a lack thereof) could also have a profound impact on autistic women’s sense of identity. The aspects of the diagnostic process which can have a negative impact on autistic women’s identity have been found to include general practitioner’s (GP’s) dismissing concerns,^12^ and an historic lack of diagnostic availability and information surrounding autistic women’s diagnoses.^8^ In addition, Bargiela et al.^12^ found that masking, both pre- and post-diagnosis, can create confusion for autistic women surrounding their identity. Although many women report relief and positive feelings at diagnosis, identity can be negatively impacted due to self-doubt and feelings of grief for autistic women.^13^ However, this study also found that many women report a positive impact of diagnosis on their identity, due to an increase in self-acceptance and self-reflection post-diagnosis. This can in turn have a positive impact on their well-being due to an increased understanding of themselves, their behaviours, and their interpersonal relationships. One area which has supported autistic women to form their identity is social media; Bargiela et al.^12^ found that through connecting with other autistic women, participants were able to form connections and identities based on their special interests rather than traditional societal norms for women.

In sum, previous research documents important relationships between diagnosis, well-being, and identity in autism women. This study aimed to investigate these relationships further, by considering these issues in women at different stages of diagnosis, specifically: those who have not been diagnosed but who self-identify as autistic, those in the process of being diagnosed or who have recently been diagnosed, and those who are several years post-diagnosis. By comparing these groups on measures of well-being and interviewing them about their identities in relation to their diagnostic status, we aimed to explore how the process of diagnosis impacts autistic women, their well-being, and identities.

## Methods

### Positionality statement

The research team for this study was neurodiverse, with one autistic researcher and two non-autistic researchers. The researcher conducting the interviews was autistic and disclosed this to participants before the start of each interview. In addition, the autistic researcher completed the first round of coding which was then verified by the rest of the research team.

### Design

A mixed-methods design was used for this study. Quantitative data were collected through surveys measuring autistic traits, quality of life, and autism-related quality of life, and individual characteristics (age, diagnostic stage, and gender identity). Semi-structured interviews were then conducted, using information from the surveys to inform interview schedule design, allowing participants to expand on their experiences and explore the more specific details of these. Data collection took place more than 8 weeks in the summer of 2021 for both the questionnaires and interviews.

An a priori power analysis for the quantitative portion was conducted using G*Power 3.1.9.7^14^ which indicated that the minimum sample size required to achieve 80% power for detecting a medium effect^15^ at a significance criterion of α = .05 was 42 for a one-way analysis of variance (ANOVA). Therefore, the researchers set a target of 50 questionnaire participants to gain sufficient power, and 20 interview participants to gain a rich data set.

### Participants

Participants were recruited via the researchers’ social media channels where adverts were posted. Overall, 96 women completed the questionnaire surveys, and 24 of these women agreed to also take part in an interview. The inclusion criteria were that the participant identified as women, had gained a diagnosis in the United Kingdom or identified as autistic and were living in the United Kingdom, and were above 18 years of age. The ages of interview participants were not recorded to ensure their anonymity and that no links were made between individual participants’ questionnaire and interview data. Table 1 shows the sample characteristics of online questionnaire participants, and their age at diagnosis was not recorded or whether the participants in Group 2 were undergoing assessment or recently diagnosed.

**Table 1.** Sample characteristics of online questionnaire participants.

Age (years)	Self-identifying/awaiting assessment	Undergoing assessment/less than a year post-diagnosis	More than a year post-diagnosis	Total
18–24	9	0	4	13
25–34	11	12	16	39
35–44	5	7	9	21
>45	12	4	7	23
Total	37	23	36	96

Participants were asked at what stage of their diagnostic journey they were at: Group 1 = self-identifying or awaiting assessment (interviews n = 8); Group 2 = undergoing assessment/less than a year since diagnosis (interviews n = 5); and Group 3 = more than a year post-diagnosis (interviews n = 11). Of the interview participants who were in Groups 2 and 3, 5 participants were diagnosed privately within a setting separate from the British National Health Service (NHS) and 11 on the NHS free of charge, and 14 participants were diagnosed as adults with only 2 diagnosed in childhood. The age at diagnosis did not appear to lead to a difference in experience for the women in this study, the important factor appeared to be the time of acceptance/understanding of the diagnosis which for all women was in late adolescence/adulthood. Of the participants in Group 2, four had been diagnosed in the past year and one was undergoing their assessment.

A Fisher’s exact test was run on the group × age cross-tabulation and revealed that the proportion of those in different age groups was not significantly different across diagnostic status groups, suggesting that age and diagnostic status are independent variables (p = .082).

Diagnoses were not verified independently, but 90% of participants met the clinical cut-off point for diagnostic referral on the AQ-10. There was no main effect of group on AQ-10 score (F(2) = .17, p = .864), indicating that those who were not yet diagnosed had similar levels of autistic traits to those who had a diagnosis.

### Materials and procedure

Ethical approval was gained from the University of Bristol School of Education Ethics Committee prior to the commencement of data collection (Approval No. 2021-8772-8719). Participants provided separate written consent for participation in the online questionnaire and interview. Participants initially completed the online questionnaire, with the option to sign-up for an interview offered on completion.

#### Online questionnaire

Participants completed the questionnaires anonymously via their own electronic device. The questionnaire surveys were delivered through the University of York’s Qualtrics-XM (Qualtrics, 2021) and included the following demographic questions: participants’ age range, gender identity, and what stage of their autism diagnostic journey they were at. Measures of autistic traits and quality of life were also collected, in the order presented below. The questionnaires took approximately 15 min to complete.

##### Autism Spectrum Quotient (AQ-10)

This psychometric measure was used as an indicator of autistic trait level of the participants.^16^ This tool is used to assess the autistic traits of adults without a co-occurring moderate or severe intellectual disability, giving us an indication of the likelihood of an individual being autistic, with a score of 6 or more indicating high levels of traits and being the cut-off for referral for assessment. As having an autism diagnosis was not a criterion for inclusion in this study, no further measures or confirmatory tests were run surrounding this.

##### World Health Organization Quality of Life short version (WHOQoL-BREF)

The WHOQoL-BREF was used in this study as a measuring of overall well-being through the perception of participant’s quality of life (QoL) reported in the survey.^17^ The test has 26 questions measuring: overall QoL, overall satisfaction with health, physical health, psychological health, social relationships satisfaction, and environmental health.

##### Autism Spectrum Quality of Life

Used alongside the WHOQoL-BREF, this survey pays more specific attention to factors which have been shown to specifically impact the well-being, and QoL of autistic individuals.^18^ The questionnaire specifically looks at the participant’s perception of support, friendships, barriers faced, and satisfaction with their identity as autistic.

#### Interviews

Individual interviews were conducted by an autistic member of the research team with each participant over Zoom (Zoom Video Communications Inc., 2016) as it was not possible to conduct face-to-face interviews due to the COVID-19 pandemic. Two participants chose to have their cameras off. Participants were made aware of the researcher’s positionality as autistic prior to the commencement of the interview. An interview schedule (see supplementary materials) was developed by the research team to be used as a guide and sent to participants ahead of their interview. The interviews were semi-structured, whereby the researcher followed the interview schedule as a guide, but also asked participants to elaborate where necessary or did not ask certain questions if deemed not appropriate. The interview focused on five key areas: the participant’s diagnostic experience (or anticipation thereof), their identity (or lack thereof) as an autistic woman, their well-being, the support (or lack thereof) they had, and any factors which were beneficial or barriers to them receiving a diagnosis. The interviews took place in July and August 2021 and lasted between 20 and 50 min (mean = 36 min). Following the interview, participants were provided with the option to receive a copy of their transcript and given the opportunity to email the researchers with any comments on the transcript or any further thoughts they had post-interview. Three participants responded with written feedback on their transcript or further thoughts, and these were incorporated into their transcripts for analysis. Interview participants each received a £20 Amazon voucher. The audio from each Zoom interview was recorded, and a transcript automatically generated by Zoom which was reviewed and corrected by the interviewer.

### Data analysis

Quantitative data analysis was completed using IBM SPSS (version 27.0), while qualitative data analysis coding was completed using NVivo (version 12). Interview transcripts were analysed by applying inductive reflexive thematic analysis.^19^ Data were analysed in six steps following Braun and Clarke’s^19^ approach: 1. familiarization with the data; 2. initial code generation; 3. collation of codes into potential themes; 4. revision of themes through discussion between authors; 5. refining and naming of themes; 6. production of report. Initially, the first author coded one transcript from each group (overall 12.5%) before sharing the transcript and coding with the other authors for feedback. The first author coded the remaining transcripts and generated initial themes, and then the entire team met to discuss the generation of themes and a thematic structure. Saturation was achieved with the data set of 24 interviews as the last two interviews coded in each group (total n = 6) generated no new codes.

## Results

### Online questionnaires

Table 2 includes the descriptive statistics for the questionnaire measures. For all questionnaires, higher scores indicate higher levels of autistic traits or quality of life.

**Table 2.** Questionnaire measure descriptive statistics by diagnostic stage group.

Measure	M (SD) Range			
	Overall	Group 1 (n = 37)	Group 2 (n = 23)	Group 3 (n = 36)
AQ-10	7.77 (1.53) 4–10 (0–10)	7.68 (1.56) 4–10	7.91 1.44 5–10	7.78 (1.59) 4–10
WHOQoL	212.3 (47.62) 101–328 (0–410)	197.56 (54.03) 101–328	237.65 (37.17) 146–293	211.25 (40.39) 137–277
ASQoL	2.93 (0.55) 1–5 (0–5)	2.92 (0.58) 2–4	3.12 (0.43) 2–4	2.81 (0.57) 1–4

AQ-10, Autism Spectrum Quotient; WHOQoL: World Health Organization Quality of Life short version; ASQoL: Autism Spectrum Quality of Life.

**Figure 1.**
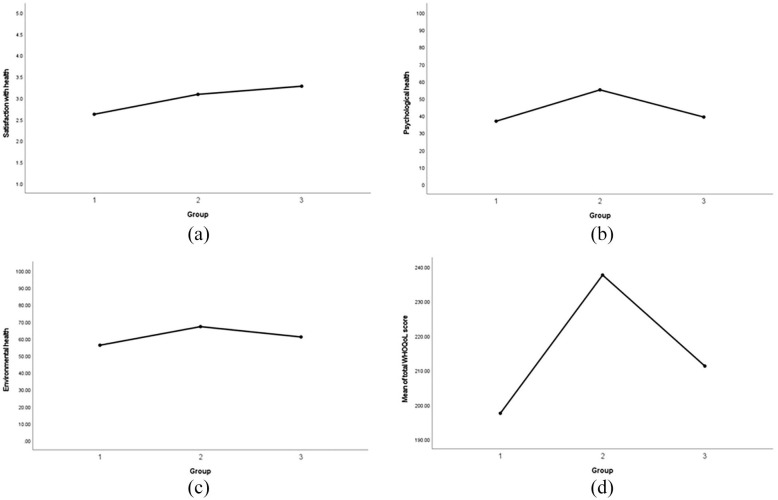
The patterns of effect across groups: (a) satisfaction with health, (b) psychological health, (c) environmental health, and (d) total score.

A one-way ANOVA revealed there were significant main effects of diagnostic stage group on the following measures from the WHOQoL-BREF Table 3: satisfaction with health (F(2) = 4.56, p = .013), psychological health (F(2) = 9.39, p < .001), and environmental health (F(2) = 4.58, p = .013). The patterns of these effects are seen in Figure 1. There were no significant main effects of diagnostic stage group on overall quality of life, physical health, or social relationships satisfaction. The one-way ANOVA also revealed a significant main effect of diagnostic stage group on total WHOQoL score (F(2) = 5.52, p = .005). Follow-up analysis using the Bonferroni test showed that the mean scores for satisfaction with health increased with each diagnostic stage, whereas the mean scores for psychological health, environmental health, and total WHOQoL score were highest in Group 2, followed by Group 3, with Group 1 having the lowest mean scores (Figure 1). In the Autism Spectrum Quality of Life (ASQoL), a one-way ANOVA revealed no significant main effect of diagnostic stage group on overall score or autistic identity score.

**Table 3.** Descriptive statistics for WHOQoL and ASQoL measures by diagnostic stage group.

	Diagnostic stage group	M (SD)		
		1	2	3
WHOQoL	Total WHOQoL	197.56 (54.03)	237.65 (37.17)	211.25 (40.39)
	Satisfaction with health	2.62 (.98)	3.09 (.95)	3.28 (.91)
	Psychological health	36.82 (20.11)	55.07 (14.10)	39.24 (13.92)
	Environmental health	56.17 (14.65)	67.12 (12.50)	61.03 (13.35)
	Overall QoL	3.62 (.86)	3.83 (.58)	3.44 (.74)
	Physical health	49.90 (14.98)	57.45 (11.19)	54.96 (14.52)
	Social relationship satisfaction	48.42 (19.72)	51.09 (18.69)	49.31 (17.29)
ASQoL	Total ASQoL	2.93 (.58)	3.12 (.43)	2.81 (.57)
	Autistic identity	3.70 (1.12)	3.7 (1.30)	3.83 (1.18)

WHOQoL: World Health Organization Quality of Life short version; ASQoL: Autism Spectrum Quality of Life.

### Interviews

The overarching theme of ***validation*** was developed with four subthemes: *Don’t forget I’m autistic, What now?, Having to be the professional*, and *No one saw me*. Additional supporting quotes for each subtheme can be found in the Supplementary Materials. The quotes are drawn from the transcripts of participants, and each quote is labelled with a participant number (PXX), and the diagnostic group they are in (Group X).

#### Validation

The majority of participants saw diagnosis as a validation of their identity, which in turn positively impacted their well-being. Having a stronger identity as an autistic woman was reported to lead to an improvement in well-being for these women as they were able to make adjustments to their environment and day-to-day life without feeling wrong or guilty about doing so. This supports the questionnaire data which suggest that psychological and environmental well-being may improve with diagnosis compared to without diagnosis. Stereotypes were seen as a barrier to diagnosis, and to validation, with some participants reporting ‘imposter syndrome’ and worsened well-being due to not fitting the stereotypical image of autistic males:

My idea of autism was Rain Man, it was the little boy with trains and maths and so on, and I think probably as most people’s idea at the moment. (P13, Group 2)

Others had doubts that they were ‘being silly’ or ‘making it up’ due to feeling they had previously been gaslit by, or lacked validation from, healthcare professionals. Diagnosis was also seen as validation for these women as it allowed them to access support; prior to diagnosis, they did not have ‘a piece of paper’ which validated them as autistic, meaning access to support was far more limited (if not impossible). Gaining this validation removed these doubts and improved their well-being through confirming their sense of themselves as autistic:

I went to my GP, and I didn’t really know what say, but I just knew something wasn’t OK, and something needed to stop, and . . . he started off saying, because I go to work every day, that means everything’s fine and to me that wasn’t really true, and I think maybe because of my autism, I found it really hard to not go to work every day . . . (P22, Group 3)

I guess for me it’s [diagnosis] that kind of validation I’m after of this identity that I found for myself, I kind of feel like I want it, I want it to feel sort of more legitimate if that makes sense. (P08, Group 1)

However, some participants reported feeling they did not need a diagnosis, or that they had had these feelings previously, but that they felt validated by exploring their identity more and interacting with the autistic community on social media. Interaction with the autistic community was a validating experience for many of the participants, and improved their well-being, especially prior to their diagnosis, as they felt less alone in their experiences:

I’ve spoken to you know couple of late diagnosed women who said, ‘well, we lived, we lived our lives up until this point, like we, like we fought quite a lot, like you know we’ve gone against you know, all these kind of odds, and we’re here like you know how awesome are we’, and it’s like actually yeah and it’s just so positive, I think, and I think that really helps in the self-identification because you don’t you know want to identify with anything you know too negative. (P01, Group 1)

While interacting with other autistic people helped to create a positive connection with autistic identity, some participants in Groups 1 and 2 were undermined by the deficit-based model of psychiatric assessment, and this reduced their well-being due to having to work to find ways to refocus their identity away from only negatives presented in traditional clinical narratives:

When I got my report back from my diagnostic process and that was like literally 15 pages of deficits, and I felt really low for a couple of days after that, and I had to kind of really consciously kind of re-find the positivity. (P13, Group 2)

Validation also came for some participants in the form of understanding themselves and their experiences; participants reported that having an autistic identity or diagnosis allowed them to be kinder and more accepting towards themselves and their difficulties. Furthermore, in regard to stereotypes, many participants also reported the diagnosis to be validating as it relieved their feelings of failing as a ‘stereotypical woman’:

. . . before I just felt like I was a crumbling mess and I just needed to pull myself together, and I don’t have that voice myself anymore either you know telling myself oh I should be, should be doing better or I should be stronger and tougher and just sort of except like I’m going to cry over really stupid things. (P17, Group 3)

#### Don’t forget I’m autistic

The extent to which participants felt the diagnostic process and any subsequent support services were autism-friendly varied across our participants: some participants described themselves as having traits which they felt were forgotten or ignored even by those who they had disclosed their autistic identity/diagnosis to:

she sort of repeatedly said things that indicated that she didn’t mind if I behaved odd and she said, that was her thinking that she was being welcoming and inclusive; I don’t mind if you’re uncomfortable, I don’t mind if you look weird because you’re uncomfortable, that’s fine you be weird I’m not going to change anything, because I know I’m absolutely fine. (P23, Group 3)

Others noted instances where they felt particularly welcomed as an autistic person:

. . . it was just like ‘Oh, they really understand my needs’ because they had photos and biographies of everyone you were likely to meet, they had photos of all of their consulting rooms, a map, a photo of the outside of the building, so it was as if they anticipated all of the things that were likely to worry me. (P13, Group 2)

Some things described by participants reflected the interaction between autistic traits and diagnostic experiences. For example, feeling the need for ‘definitive’ validation of their identity by a professional, which could be linked to having inflexible thinking styles. When this was not made clear by the professional, this led to confusion around their autistic identity, which had a negative impact on well-being.

The wording was something like you know she meets the criteria for a positive diagnosis, should this be something that she wants, you know, so it was and I, and I talked about this at length with the psychologist like over the following couple of years, because I thought I felt uncomfortable about this idea of it was my choice . . . I’d sort of seen the diagnostic process as kind of drawing a line under that and going, you are, or you aren’t. (P15, Group 3)

Equally, finding it difficult to adapt to having a new identity as ‘confirmed’ autistic may be linked to dislike of change and intolerance of uncertainty, such as around how people will react to this information:

It’s kind of a bit of a struggle, there’s a lot of anxiety and there’s a lot of uncertainty as to how I’m going to navigate the world with this new identity. (P09, Group 2)

Furthermore, participants reported the impact of masking on their recognition, diagnosis, and validation; participants told us of the difficulties they faced with assessments due to facing the dilemma of having to ‘unmask’ to be diagnosed, which at times felt impossible due to how intertwined their true persona had become with their mask, or they would continue to mask, and the assessor would not recognize their autistic traits:

I sort of anticipate that because I’ve gotten fairly good at sort of presenting myself as a somewhat articulate and competent adult, that I’m not going to be taken seriously, and I’m not going to be able to convince people of the struggles that I do have. It feels like quite a scary thing. (P08, Group 1)

Further barriers and positive factors when seeking support for autistic women were reported around communication and practicalities: especially since due to COVID healthcare in the United Kingdom was moved to phone and video call appointments. This brought some benefits as women did not have to travel and negotiate public transport to attend appointments. However, for many of the participants, appointments became inaccessible, or access was restricted due to anxieties and difficulties in communicating when using the phone or video call. This negatively impacted their well-being, both psychologically and in some cases physically, as access to care was more difficult:

Video chatting like this, I find okay, but like on the phone I really struggled with and it kind of felt like because you couldn’t get, see anyone face to face, it was like I then couldn’t even talk on the phone because I, that was like a barrier for me. (P24, Group 3)

#### What now?

Participants from all groups discussed the long waiting times (6 months to more than 3 years) between referral and assessment, especially for those undergoing assessment through the NHS. For some, this gave them time to learn about autism and work out what being autistic meant to them, but overall, delays were linked to worse well-being for participants:

I don’t feel valid [to ask for support] because I haven’t got the formal label diagnosis and stuff and a lot of the time when you say I’m awaiting diagnosis it’s like ‘no, you need formal diagnosis’ and it’s like I can’t get that yet – that’s a nightmare. (P06, Group 1)

In addition, participants from all groups reported a lack of clarity in the diagnostic and support pathways for autistic adult women. Many participants felt like there was no clear pathway for them to follow from initially thinking about being autistic to discussions with a GP, and onto diagnosis, and that even post-diagnosis there was a lack of clarity as to where they could access support, if any:

I got diagnosed and then I had like one post-diagnostic session . . . It would have just been nice I don’t know just to, like I don’t know I felt like I was diagnosed and then like waved off on my way as, like it’d be nice if I could have just had like a couple more sessions, just like talk it through and just like dunno, process it with somebody. (P14, Group 3)

Furthermore, participants reported that even though there were many positives to identifying or being diagnosed as autistic, the experience of going back over old memories and past experiences with a new ‘autistic lens’, both pre- and post-diagnosis, was a traumatic one. Several participants discussed feeling the need to ‘unpack their past’ and that this had to be done without support. This depicts the participants’ feelings that there is not a smooth journey after identifying/diagnosis, it is not simply a matter of ‘taking off the mask’ and moving on with life as an autistic woman, but that there is still work to be done and that the experience can be traumatic and positive. For some participants, post-diagnostic support with other autistic people was helpful to combat some of these negative effects of the diagnostic process:

So, yes diagnosis: useless, in fact damaging. But the group, post-diagnosis group: brilliant, can’t recommend it enough, I think that should be mandatory, you don’t just spin people off and leave them with this bit of paper that may or may not be accurate. (P12, Group 2)

Relating to the statistical results, participants from Groups 1 and 3 reported a feeling of growing into their identity, unlike Group 2. This could suggest one reason behind the pattern seen in the psychological well-being graph, whereby during pre-diagnosis waiting or while beginning to self-identify, there is still a feeling of uncertainty which lowers well-being, then when a diagnosis is gained there is a lot of optimism, and participants feel they have found their identity, but as time passes the women feel that they still are growing into their identity, which links with the reports of unpacking and going through traumatic experiences due to that come from Group 3.

#### Having to be the professional

Many of the women reported having to do their own research to learn about autism, where to get support, where and how to seek a referral for diagnosis, and their rights. Some people report enjoying aspects of this research and learning about autism more generally; however, this theme encompasses the more negative reports of having to do research due to professionals lacking knowledge:

Yeah, so basically, I went to the doctors and I’d already researched a national autism website like how you should go to the doctors, so I printed off all the information because, like I also know that I’m quite a competent, I can come over as quite a competent person, so I printed off all of the information and took it to the doctors and said ‘I want to have it, an assessment for autism, this is all the reasons why this is the information from National Autistic Society, this is how you should refer me’, so I even told them like what my local area’s process was and, and then she said okay I’ll refer you on to the next step. (P11, Group 2)

it feels very much like the onus is on you as an individual to make the assessment happen, rather than a medical practitioner saying ‘this is something that we should see’ or we should, you know just anything any kind of update, or any kind of knowledge that they sent you on a referral and you’ve not heard anything back. (P03, Group 1)

Furthermore, participants from Groups 1 and 3 reported feeling a need to educate the wider population and be ‘a professional’ in the field in this sense too, due to feeling a lack of understanding and that there is still discrimination against autistic individuals. This led to reports of not wanting to disclose or share their identity for some of the participants:

When I interviewed for a job, I didn’t tell them because I was worried that it would impact on that, and I kind of wish I had told them . . . I think it might have helped me with better support, but at the same time, I was just so worried about like being turned down for it because I went for a volunteer job once and put on that I was on the autistic spectrum and I got turned down, and I do think it was because of that. So, sometimes when I’m comfortable with people I tell them that I’ve got the diagnosis, and then like in job interviews I won’t tell them until it’s like needed, they need to know. (P24, Group 3)

This reluctance could help to explain the survey results seen in the psychological and environmental health categories; that Groups 1 may struggle more with feelings of needing to educate others due to beginning to consider autism, and Group 3 due to being diagnosed for some time, and their struggles around disclosure. In addition, those in Groups 1 and 3 reported a lack of follow-through on discussions with professionals, suggesting that support into getting into and out of the diagnostic process is when people feel they lack professional support, compared to directly when they are getting the diagnosis:

I think it would have been good to have signposting from one agency to another . . . but nobody ever said that, so I just had to like Google, I was asking my counsellor and asking all these other people, and it just, it’s so exhausting. (P01, Group 1)

In summary, most participants across the groups reported experiences of being disbelieved and dismissed by healthcare professionals, lacking support from healthcare professionals, and even when support was given that many professionals lacked knowledge of autism and how to support an autistic person:

I can do that a lot better than I used to. I’m sure, like the therapist would want to be like oh yeah that’s because of me, and you know or the psychiatrist, but I think honestly, I’ve done a lot of the work myself. (P17, Group 3)

#### No one saw me

The majority of women interviewed reported a lack of support throughout their life, from childhood through to adulthood, especially in school; many reported being bullied, not fitting in, not being supported as people thought there was ‘nothing wrong with them’ during their school years, and some specify that they believe school to be where they started masking. Some participants also reported that they slipped into the character of a ‘good girl’ at school as this is how they were labelled by teachers. Masking for many participants was described as the reason as to why no one ‘saw them’ and supported them, then or now, whether they were aware of it at the time or not:

I think it was definitely not picked up because I was a woman . . . I’m very high functioning, I think people think that I’m like making an excuse or I’m like making it up . . . I do feel a bit let down sort of for my whole adult life I’d been told that I was mentally ill and actually no that wasn’t the case I was perfectly healthy. (P04, Group 1)

In terms of visibility, many women reported a lack of autistic women portrayed in the media. However, when autistic women were seen in the media, this was beneficial to participants with several reporting this was how they first began identifying traits in themselves:

Everyone seems to have that kind of image of like the typical autistic person is like Sherlock Holmes or Sheldon Cooper, and if you don’t identify with that, you’re probably not autistic . . . it’s not actually, as you know, black and white as the media might make it out to be. (P09, Group 2)

Multiple participants reported that professionals who did understand or recognize were those with lived experiences of autism themselves (either being autistic themselves or having an autistic family member):

At the end, she told me she said, like I’m certain that you’re autistic and then she told me that her daughter is and she’s like you, like the way you are reminds me so much of her, so I think that’s why I felt understood by her. (P14, Group 3)

## Discussion

The aim of this study was to investigate autistic women’s diagnostic experiences in relation to their identity and well-being. Overall, our results suggest that diagnosis, identity, and well-being are closely interlinked. These results partially support other work showing interactions between diagnosis, identity, and well-being for autistic women, but importantly we highlight for the first time that these effects change throughout the diagnostic process: it is not the case that well-being automatically improves with time, but that validation through diagnosis, understanding, and acceptance impacts well-being and identity the most. Getting a diagnosis appears to improve the aspects of well-being and identity at the point of diagnosis through providing validation and the provision of some answers, but longer-term women can struggle with their identity as autistic and with accessing support, which is supported by the results of the quantitative questionnaires showing psychological and environmental health do not increase linearly along the diagnostic pathway. As all but one of our interview participants were diagnosed/seeking diagnosis in adulthood, this study mostly provides evidence for the impact of diagnosis on adult autistic women’s identity and well-being.

Many of the difficulties with diagnosis, identity, and well-being reported in this study surrounded the health and social care provided, or the lack thereof. This can be seen both in the interview data and in the low scores for health-related well-being from the online surveys. This supports other reports that clinicians involved in the diagnostic process are often not adequately trained or knowledgeable about autism in women.^5^ Participants in this study reported that this leads to them feeling unseen and as though they must be the professional themselves, thereby risking the validation they are seeking – which negatively impacts well-being and identity.

As research has shown that many UK services fail to provide timely assessments,^20^ it is unsurprising that waiting times were some of the most commonly reported barriers for the women in this study when seeking a diagnosis, and that these could lead women to seek a private assessment as they felt they needed the ‘answer’ sooner than the NHS could provide. The process for seeking and gaining and assessment in other countries in private or public services may differ to the United Kingdom; therefore it is important to bear in mind these results may not be generalizable across countries and services.

This project was the first to use mixed-methods approaches to provide evidence about the role of diagnosis in autistic women’s well-being. However, we note several limitations to the current work that will need to be addressed in future projects. Our statistical tests were conducted on a relatively small sample. Our sample was British women: this means that we are missing important experiences from women of other nationalities. Indeed, reports have shown that non-British black and Hispanic autistic children are diagnosed on average later that white autistic children^21^ despite showing similar clinical profiles:^22^ this suggests that non-Caucasian autistic people may similarly not fit an autism stereotype, leading to delays in referral and diagnosis. Non-Caucasian autistic women may thus be at double risk of being missed, due to their ethnicity and their gender. In addition, due to the methods of data collection, the study focused on the reflections of autistic women who were able to respond to a written questionnaire, and for the interviews were verbal. Therefore, the experiences and reflections of those who could not read, had a severe intellectual disability, or were non-verbal were missed. In future, research should aim to further this study to include non-binary individuals as non-conformity to a binary gender may further impact an individual’s sense of identity and need for validation.

Examining the extent to which participants felt they identified as autistic might be something for future research to consider: we did not include this in our quantitative study, but the extent to which individuals feel strongly connected to an autistic identity could vary, and the strength of this identity could have relationships to well-being.^23^ Asking participants to quantify the strength of their identification and validation could also allow for studies to examine how these factors change over time: this study used a cross-sectional design but longitudinal work that follows women through their diagnostic journeys would also allow for future examination of how diagnosis impact identity and well-being over time.

Notwithstanding these limitations, our report has several implications for practice. First, reduced waiting times and clearer diagnostic pathways for autistic adults, especially women, are needed. Second, health care professionals should be mindful of the impact they can have on autistic women’s feelings of validation. For example, while it is common to write a diagnostic report that details the deficits individuals showed, which are used to confirm the presence of autism, several women found these reports to be discouraging.

This may be helped in part by our third recommendation, that greater post-diagnostic support is offered: this may help to mitigate some of the negative impacts of reading a diagnostic report that documents one’s shortcomings. We would recommend further work to examine when the timing of this post-diagnostic support is most suitable. A single post-diagnostic session to discuss diagnosis shortly after it is given may be too soon, and given that women’s well-being appears to peak shortly after diagnosis and then wane over time – a year or more after diagnosis – it may be suitable to consider whether support after a year could be offered. This could include some trauma focused^24^ and identity building work.

Finally, training on the presentation of autism in women and girls for primary and secondary health care professionals is needed. Participants reported experiences of being told they were likely not autistic because they were working or because they had not been identified as autistic as a child: assumptions such as these need challenging, as it suggests health care professionals may assume that because autism is a lifelong disorder that all cases would have been detected in childhood. This misses the point that for some individuals, they are able to cope and mask their autistic difficulties into adulthood, when they nonetheless would benefit from diagnosis and support.

## Conclusion

In conclusion, we found that autistic women’s well-being and identity interacted with the level of validation they felt of said identity by themselves and others, specifically healthcare professionals. Validation was seen to be associated with diagnosis for many of the women, suggesting initially that well-being and identity for autistic women are improved with diagnosis. However, well-being and identity were negatively impacted for autistic women due to a lack of support, both pre- and post-diagnosis, from health and social care professionals; long waiting times, lacking post-diagnosis care or support, and insufficient knowledge on the professionals’ part are reasons for this. Future work should aim to quantify autistic women’s feelings of validation and correlate these with quantified measures of autistic identity strength and well-being to explore this further.

## Supplemental Material

sj-docx-1-whe-10.1177_17455057221137477 – Supplemental material for Autistic women’s diagnostic experiences: Interactions with identity and impacts on well-being Click here for additional data file. Supplemental material, sj-docx-1-whe-10.1177_17455057221137477 for Autistic women’s diagnostic experiences: Interactions with identity and impacts on well-being by Miriam Harmens, Felicity Sedgewick and Hannah Hobson in Women’s Health

sj-docx-2-whe-10.1177_17455057221137477 – Supplemental material for Autistic women’s diagnostic experiences: Interactions with identity and impacts on well-being Click here for additional data file. Supplemental material, sj-docx-2-whe-10.1177_17455057221137477 for Autistic women’s diagnostic experiences: Interactions with identity and impacts on well-being by Miriam Harmens, Felicity Sedgewick and Hannah Hobson in Women’s Health

sj-docx-3-whe-10.1177_17455057221137477 – Supplemental material for Autistic women’s diagnostic experiences: Interactions with identity and impacts on well-being Click here for additional data file. Supplemental material, sj-docx-3-whe-10.1177_17455057221137477 for Autistic women’s diagnostic experiences: Interactions with identity and impacts on well-being by Miriam Harmens, Felicity Sedgewick and Hannah Hobson in Women’s Health

## Declarations

**Ethics approval and consent to participate:** The project received ethical approval from the University of Bristol School of Education Ethics Committee. All participants gave fully informed consent to participate.

**Consent for publication:** All participants gave explicit consent for their data to be used in publications.

**Availability of data and materials:** Data and materials are available on request from FS (felicity.sedgewick@bristol.ac.uk) upon reasonable request.
