# Supplementary material for: Autistic women’s diagnostic experiences: Interactions with identity and impacts on well-being
Source: Womens Health (Lond). 2022 Nov 15;18:17455057221137477. doi: 10.1177/17455057221137477 (PMC9666868; doi:10.1177/17455057221137477)
Supplement: sj-docx-1-whe-10.1177_17455057221137477 – Supplemental material for Autistic women’s diagnostic experiences: Interactions with identity and impacts on well-being [file sj-docx-1-whe-10.1177_17455057221137477.docx]

| **Domain 1: Research team and reflexivity** |  |  |  |  |
| --- | --- | --- | --- | --- |
| Personal Characteristics |  |  |  |  |
| 1. | Interviewer/facilitator | Which author/s conducted the interview or focus group?  Miriam Harmens  As mentioned in the paper this author is autistic and disclose this at interviews to the participants |  |  |
| 2. | Credentials | What were the researcher's credentials? *E.g. PhD, MD*  Miriam Harmens - BSc  Felicity Sedgewick – PhD  Hannah Hobson - PhD |  |  |
| 3. | Occupation | What was their occupation at the time of the study?  Miriam Harmens – Student  Felicity Sedgewick – Lecturer  Hannah Hobson - Lecturer |  |  |
| 4. | Gender | Was the researcher male or female?  All researchers are female |  |  |
| 5. | Experience and training | What experience or training did the researcher have?  Miriam Harmens – worked with autistic individuals in social care for several years and received informal training on interview conducting and research design and analysis as well as completing degree at the time of research  Hannah Hobson and Felicity Sedgewick – both have several years experience both working with autistic people and conducting research with this and other clinical groups. |  |  |
| Relationship with participants |  |  |  |  |
| 6. | Relationship established | Was a relationship established prior to study commencement?  None beyond that required for organising participation |  |  |
| 7. | Participant knowledge of the interviewer | What did the participants know about the researcher? e*.g. personal goals, reasons for doing the research*  They knew that the researcher conducting the interviews was also autistic and that the research was being conducted with the aim of expanding the knowledge base, giving a voice to autistic women, and improving services in the future |  |  |
| 8. | Interviewer characteristics | What characteristics were reported about the interviewer/facilitator? e.g. *Bias, assumptions, reasons and interests in the research topic*  It was reported in the paper that the team is neurodiverse, with an autistic researcher conducting the interview.  This was included to make it clear that participants were fully informed about the understanding the interviewer had of their experiences. |  |  |
| **Domain 2: study design** |  |  |  |  |
| Theoretical framework |  |  |  |  |
| 9. | Methodological orientation and Theory | What methodological orientation was stated to underpin the study? *e.g. grounded theory, discourse analysis, ethnography, phenomenology, content analysis*  Reflexive thematic analysis (Braun and Clarke, 2006) as this allowed for data-led analysis, rather than following a priori assumptions |  |  |
| Participant selection |  |  |  |  |
| 10. | Sampling | How were participants selected? *e.g. purposive, convenience, consecutive, snowball*  Opportunistic sampling using advertising on the researcher’s social media |  |  |
| 11. | Method of approach | How were participants approached? e*.g. face-to-face, telephone, mail, email*  The questionnaire advert was posted on social media, which participants completed an online questionnaire without contact with researcher. For those who opted in for interview: contact was maintained via email, and interviews were all conducted via zoom (with optional video) |  |  |
| 12. | Sample size | How many participants were in the study?  96 online questionnaire participants, and 24 of these completed an interview |  |  |
| 13. | Non-participation | How many people refused to participate or dropped out? Reasons?  None |  |  |
| Setting |  |  |  |  |
| 14. | Setting of data collection | Where was the data collected? e*.g. home, clinic, workplace*  Via zoom – as far as researcher’s were aware all participants connected to zoom from home. |  |  |
| 15. | Presence of non-participants | Was anyone else present besides the participants and researchers?  No one (some participants had children in the house, but none interrupted) |  |  |
| 16. | Description of sample | What are the important characteristics of the sample? *e.g. demographic data, date*  Identified as female  Over 18  From the UK  Identified as autistic  Data collected in July and August 2021 |  |  |
| Data collection |  |  |  |  |
| 17. | Interview guide | Were questions, prompts, guides provided by the authors? Was it pilot tested?  Prior to the interview, those participants who indicated that they would benefit from seeing the interview schedule were sent it |  |  |
| 18. | Repeat interviews | Were repeat interviews carried out? If yes, how many?  None |  |  |
| 19. | Audio/visual recording | Did the research use audio or visual recording to collect the data?  Audio and visual recording via zoom |  |  |
| 20. | Field notes | Were field notes made during and/or after the interview or focus group?  No |  |  |
| 21. | Duration | What was the duration of the interviews or focus group?  Interviews lasted from 15 minutes to 45 minutes depending on the length of participants’ answers |  |  |
| 22. | Data saturation | Was data saturation discussed?  Yes – no new themes emerged in the last two interviews for each group within the study. This is presented in the paper. |  |  |
| 23. | Transcripts returned | Were transcripts returned to participants for comment and/or correction?  Participants were offered this, though few requested to see the transcript and some actively said they did not want to see it. Transcripts were returned to those who requested, and only one was returned to interviewer with comments which were incorporated into the analysis |  |  |
| **Domain 3: analysis and findings** |  |  |  |  |
| Data analysis |  |  |  |  |
| 24. | Number of data coders | How many data coders coded the data?  Three |  |  |
| 25. | Description of the coding tree | Did authors provide a description of the coding tree?  This is not included in the paper as the coding process and discussions did not produce a formal coding tree |  |  |
| 26. | Derivation of themes | Were themes identified in advance or derived from the data?  Derived from the data |  |  |
| 27. | Software | What software, if applicable, was used to manage the data?  NVivo |  |  |
| 28. | Participant checking | Did participants provide feedback on the findings?  Participants were sent a study summary following initial analysis and had the opportunity to comment – only comments were positive and in agreement with the interpretation of their interviews |  |  |
| Reporting |  |  |  |  |
| 29. | Quotations presented | Were participant quotations presented to illustrate the themes / findings? Was each quotation identified? e*.g. participant number*  Yes, participants were identified via a participant number and group number |  |  |
| 30. | Data and findings consistent | Was there consistency between the data presented and the findings?  Yes |  |  |
| 31. | Clarity of major themes | Were major themes clearly presented in the findings?  Major themes are summarised individually in the findings and presented in a thematic map |  |  |
| 32. | Clarity of minor themes | Is there a description of diverse cases or discussion of minor themes?  Examples from specific quotes were used to display diversity within themes and what each theme fully encapsulated |  |  |
