# Supplementary material for: Autistic women’s diagnostic experiences: Interactions with identity and impacts on well-being
Source: Womens Health (Lond). 2022 Nov 15;18:17455057221137477. doi: 10.1177/17455057221137477 (PMC9666868; doi:10.1177/17455057221137477)
Supplement: sj-docx-2-whe-10.1177_17455057221137477 – Supplemental material for Autistic women’s diagnostic experiences: Interactions with identity and impacts on well-being [file sj-docx-2-whe-10.1177_17455057221137477.docx]

Interview questions

Please note that the interviews we aim to conduct will be semi-structured, meaning we may add in questions, change the order, or not ask questions on the day as we feel relevant. This document is designed to give you an idea of the type of questions we are hoping to ask in the interviews.

Firstly, how long have you been self-identifying as auitsic and/or diagnosed for?

When did you start thinking you had autism?

At what point did you really begin to identify as autistic?

IF RELEVANT: Could you please tell me about the diagnostic process you went through, what happened and how did it go?

OR: Could you please tell me about your experience of the process to try and get diagnosed so far, what has happened and how is it going?

OR: Do you want to undergo the diagnostic process and if so what are your thoughts/feelings on this?

Do you consider yourself to have an autistic identity, and if so would you please describe this to me?

Has your identity as an autistic person changed along your diagnostic journey at all, and if so at what point or points?

Do you feel that having a diagnosis would change/has changed your identity as an autistic person, and if so how?

How do you feel having a diagnosis would impact/does impact society's view of your identity as an autistic person?

Do you feel that being a woman can have an impact on your connection to your identity as autistic?

How would you describe your wellbeing at this point?

How do you feel your wellbeing has changed throughout your journey to self-identifying/getting diagnosed, if at all?

If so, why do you think your wellbeing has changed like this?

Have you felt supported through your journey as an autitsic woman?

Where have you gained this support from?

If you felt unsupported at times, why do you believe this was? (could you not ask for support/were you not gaining the support you asked for and why?)

Do you believe you should have had more support, and if so from where?

Do you feel like the level of support you have received has impacted your wellbeing?

Are/were there any factors which helped you when seeking/gaining a diagnosis, and if so what do you believe these to be?

Are/were there any barriers you face/faced when seeking/gaining a diagnosis, and if so what do you believe these to be?

What impact do you believe these factors have/have had on your identity as an autistic woman?

How did these factors change your wellbeing, if at all?
