# Supplementary material for: Autistic women’s diagnostic experiences: Interactions with identity and impacts on well-being
Source: Womens Health (Lond). 2022 Nov 15;18:17455057221137477. doi: 10.1177/17455057221137477 (PMC9666868; doi:10.1177/17455057221137477)
Supplement: sj-docx-3-whe-10.1177_17455057221137477 – Supplemental material for Autistic women’s diagnostic experiences: Interactions with identity and impacts on well-being [file sj-docx-3-whe-10.1177_17455057221137477.docx]

| Theme/subtheme | Group 1 | Group 2 | Group 3 |
| --- | --- | --- | --- |
| Validation | *I guess for me it's [diagnosis] that kind of validation I'm after of this identity that I found for myself, I kind of feel like I want it, I want it to feel sort of more legitimate if that makes sense.* **P8**  *you feel empowered, but you feel powerless, at the same time; empowered because you know a bit more, but powerless because you have to wait to be diagnosed by somebody else.* **P1**  *online communities, without a doubt [helped when seeking diagnosis. Like they just really validate like everything that you're thinking, even things that you didn't know you're thinking.* **P2**  *by following people on Twitter for the last year, seeing them talk more and kind of validating what I'm feeling as being the views the, the situations that other autistic people find themselves in, especially women, and it has completely validated that that, that is the case, and I can, and I can positively self-identify as having an autistic identity.* **P3**  *I feel like it's not a label that I would want to claim for myself unless I had an official diagnosis.* **P7**  *I don't feel valid [to ask for support] because I haven't got the formal label diagnosis and stuff and a lot of the time when you say I'm awaiting diagnosis it's like ‘no, you need formal diagnosis’ and it's like I can't get that yet - that's a nightmare.* **P6**  *I've spoken to you know couple of late diagnosed women who said, ‘well, we lived, we lived our lives up until this point, like we, like we fought quite a lot, like you know we've gone against you know, all these kind of odds, and we're here like you know how awesome are we’, and it's like actually yeah and it's just so positive, I think, and I think that really helps in the self-identification because you don't you know want to identify with anything you know too negative.* **P1** | *When I got my report back from my diagnostic process and that was like literally 15 pages of deficits, and I felt really low for a couple of days after that, and I had to kind of really consciously kind of refind the positivity.* **P13**  *whereas now it's kind of like I can say ‘I'm struggling with this thing and it's because I'm autistic’ and they're not dismissing it anymore they're kind of going 'okay that makes sense', and you know, taking it with the gravity that I feel it should have and so it's yeah, a bit more validity in what I'm trying to discuss with people has been nice.* **P9**  *it's enabled me to think of myself differently, think of myself as not being kind of like really Princessy because I can't bear the noise and things like that, to feel that my needs, my peculiar needs are valid, and I've been able to ask people…to do things about it.* **P13**  *so now it's like 'Okay, no I don't want to go to that situation and that's totally fine because my brain needs a rest' or 'yeah, it's fine to just like absolutely not talk to my husband for 45 Minutes because I just need to like zone out' and like and he appreciates that as well, so it's, it's a, it's really nice for me to know and yeah I can be kinder to myself, because of it.* **P11**  *I think, because, particularly because I'm going through the diagnostic process now, that actually I, I feel like I do need, not that it's external validation, but it's kind of, also confirmation isn't quite right, because if it was confirmation it would mean that I was already there, because they were confirming something that I already felt, so I don't really know what the word is, but that I, yeah until it's sort of identified and kind of seen by someone else who is an expert in these things then, I suppose, seen is kind of the closest word and, and I think even then I imagine that it will probably take a while to get you know, like it it's not going to be like ‘oh yeah there we go and now that's my identity’.* **P10**  *My idea of autism was Rain Man, it was the little boy with trains and maths and so on, and I think probably as most people's idea at the moment.* **P13** | *I think well it's not me, it's not me being a freak, there's like a reason for why I might do things…I think I just thought that I didn't have to hide it as much because I wasn't like a fake if that makes sense and I'm not saying that people who self-diagnose are fakes, I don't think that, but I just felt like that, for me, so, and I think there's loads reasons why people self-diagnose and don't get officially diagnosed, but that's a whole different story, so I just think, I just felt I didn't have to hide stuff as much because I could say, well, actually, I do have that diagnosis now.* **P21**  *there was a sense of relief that those things are my fault you're like 'it's okay' and I've been trying, you know, since my little 11-year-old self decided I will sort this out, I've been actively trying to fix those things, and that understanding that I can't do that is a relief.* **P23**  *I felt relieved hugely relieved that you know it, everything made a lot of sense, and you know that there was a reason for why I always felt different and that something wasn't quite right with me… I think it's really helped me to, to have this official confirmation that you know, I was right and it's not just something I'm making up. Yes, yes, it's definitely changed that and now I can say I can proudly identify with, with that with that label as well.* **P19**  *because it had taken so long, because I'm always very, not forceful, but that kind of thing when I'm trying to get help and assistance, I was worried that I would push them to give me the diagnosis if it wasn't real. But she was able to pull out parts of things and say ‘this is, as soon as you said this bit, I realized that you were autistic because that's a very classical thing for autistic people’. And so, and so that was, it was good, validation.* **P20**  *I think I didn't identify as autistic until after I'd been through the process and someone had said yes, because a psychiatrist saying it is a good suggestion that you probably some, something similar or autistic traits or something, but for me it was when, when the lady after the diagnosis said, said I was, that's when I was like ‘Okay, this is this is part of me and, and that's okay’… I think I would say that now that it's been diagnosed, and it's been officially told, and I have a report that says I'm autistic, and this is why, I think I probably do consider myself to have an autistic identity.* **P22**  *people who have known me like for a long time, saying like after diagnosis, they were like you just seem so much happier, and I'm like I am because I feel able to do myself now so, but I did feel like I don't know like a massive change in me; I just felt a lot more relaxed and a lot more content being myself, and like rather than like hiding parts of myself all the time, so I think that has massively like benefited my well being.* **P14**  *I think straightaway I, I knew there was something in it [autism], so in that respect I almost did [identify] straight away, but I, I couldn't take it as, until I had a diagnosis on a piece of paper, I couldn't say to myself that's me I could just say ‘Oh, I think that's probably me’, which, in my head is not sufficient to qualify me, as it were, not that you need to be qualified to be autistic but I yeah, so sort of in a way, I started identifying straightaway traits wise, but I couldn't kind of take on that identity for a while.* **P18**  *I was expecting the diagnostic journey to provide a certain clarity, I suppose a certain security that that was part of my identity.* **P15**  *for me an identity shouldn't need to be verifiable by a piece of paper, but if I have support needs which are gate kept by people who demand pieces of paper it's that way in. And so, in terms of, and it shouldn't be that way at all, in my opinion, but in terms it feels like the, the social barriers which are put in place the diagnosis actually allows me to access support which I should get anyway, so it almost is a pass through those hoops if that makes sense.* **P16**  *…before I just felt like I was a crumbling mess and I just needed to pull myself together, and I don't have that voice myself anymore either you know telling myself oh I should be, should be doing better or I should be stronger and tougher and just sort of except like I'm going to cry over really stupid things.* **P17**  *So, kind of, after sort of being diagnosed and researching more into it and talking to other people on the spectrum, it like really helped me feel a lot more like accepting of myself and, and that then took a step to being like a lot more happier with myself… it was quite a lot of relief really that like I wasn't like broken, I guess.* **P24**  *I went to my GP, and I didn't really know what say, but I just knew something wasn't OK, and something needed to stop, and… he started off saying, because I go to work every day, that means everything's fine and to me that wasn't really true, and I think maybe because of my autism, I found it really hard to not go to work every day…* **P22, Group 3** |
| Don’t forget I’m autistic | *I sort of anticipate that because I've gotten fairly good at sort of presenting myself as a somewhat articulate and competent adult, that I'm not going to be taken seriously, and I’m not going to be able to convince people of the struggles that I do have. It feels like quite a scary thing.* **P8**  *I know certainly at work because I'm quite sort of black and white about things people can sometimes take me the wrong way and I think it's just that peace of mind of sort of saying you know if I did upset someone, for whatever reason I, it's unlikely that I would, but just to be able to say, well, no, this is why and I'm sorry that I've done that, I haven't done anything like to you maliciously, it's sort of that protection from if I get into trouble for upsetting someone.* **P4**  *I think one of the interesting things about lockdown has been, my husband and I have been talking about it, and he's used to me being the sort of social butterfly out and about doing all these events and stuff like this and now I'm like I want to stay at home with my cats, I do not want to go into the back into the office, I'm not interested in going back to the office, and I think part of that is because I've realized exactly how much of an effort all of that was and how sort of reverting to my sort of natural childhood kind of shy and geeky type self does feel more natural.* **P7**  *I have these moments, where I just think I would honestly give anything, not to identify as such, because it, life can just be so difficult and so overwhelming.* **P6** | *it's been, it's kind of a bit of a struggle there's a lot of anxiety and there's a lot of uncertainty as to how I'm going to navigate the world with this new identity.* **P9**  *I think the lack of understanding from the GP did [negatively impact wellbeing], because I think it's in my nature to feel things quite acutely.* **P13**  *…it was just like ‘Oh, they really understand my needs’ because they had photos and biographies of everyone you were likely to meet, they had photos of all of their consulting rooms, a map, a photo of the outside of the building, so it was as if they anticipated all of the things that were likely to worry me.* **P13**  *it's not because like I'm like ashamed to be autistic or like it's just because I'm still fitting into my new, well I don't like change because I'm autistic, and before I didn't know, I was like I knew but I didn't, hadn't been diagnosed, so I think the process [identifying] is likely to take longer.* **P11** | *I was referred to the psychologist um and the interviews for that part of process were really fascinating because she was somebody who's very used to dealing with autistic people, very used to respecting our communication style, very used to sensory preferences, and when I walked in the chairs weren't automatically opposite each other, when I took my shoes off to sit on the chair, she didn't raise an eyebrow she, she, it wasn't even that she didn't raise an eyebrow, she didn't even consider raising an eyebrow, that was normal to her. She asked about a load of questions about the room, would you like me to make it lighter or darker is it warm enough in here for you. And then we had a conversation, where I didn't have to pretend not to be autistic and autistic communication was already respected.* **P23**  *I then went to my GP, and because I wanted, I'm a black and white, I needed I needed to know, so I went to them with a big old list of reasons why I think this is me and they looked at my list and looked at me and they said ‘Oh yes, we'll refer you’.* **P18**  *the wording was something like you know she meets the criteria for a positive diagnosis, should this be something that she wants, you know, so it was and I, and I talked about this at length with the psychologist like over the following couple of years, because I thought I felt uncomfortable about this idea of it was my choice whether or not I thought you know, because I had been in this period of you know where before the assessment where I've done all this research and had been thinking about whether it might apply to me or not I'd sort of seen the diagnostic process as kind of drawing a line under that and going, you are, or you aren't.* **P15**  *it's kind of not really due to the diagnosis, but it is related because it was the change, I was struggling with even though it's something I really wanted, it, I just couldn't deal with it, so I did decline like quite a lot and that's when you know, I was at my lowest. So, it's taken me like a good like nearly two years to kind of work myself back up to the highest that I've than I've ever been.* **P17**  *she got it set up with two chairs directly opposite each other and not even with a table in between, like our knees could touch. And I tried to sit in the chair, but I was physically squirming, and I said to her, she got the notes and she you know she hadn't looked at them, and I said to her I'm autistic I find eye contact difficult and she said that's okay I don't mind, and my husband was there, and he said afterwards like it wasn't about whether she minded or not and I, and she sort of repeatedly said things that indicated that she didn't mind if I behaved odd and she said, that was her thinking that she was being welcoming and inclusive; I don't mind if you're uncomfortable, I don't mind if you look weird because you're uncomfortable, that's fine you be weird I'm not going to change anything, because I know I'm absolutely fine. And it would have been a very simple thing to just say ‘Oh, do you want to move the Chair’, you know, but there was no thought in her that it was anything to do with her all the difficulty was, was mine.* **P23**  *Video chatting like this, I find okay, but like on the phone I really struggled with and it kind of felt like because you couldn't get, see anyone face to face, it was like I then couldn't even talk on the phone because I, that was like a barrier for me.* **P24** |
| What now? | *it's just this is actually the first time I've ever really spoken to anyone outside of my friends about it, and it's quite a, it's quite a nerve-racking thing to think about actually approaching like my GP for a referral.* **P8**  *it's really weird in between the, I think, in between being told that you've been misdiagnosed, and you have to wait for an assessment, there's this bit in the middle where lots of people I've discovered are kind of residing, where there's no help, there's no support.* **P1**  *I would go privately or maybe not even in, the NHS I don't trust, what they've done with my daughter…undermined their parenting and the way they support the children and, and it's involved with the children services and I'm really, really, really scared about that.* **P5**  *I went to my GP to ask for a referral through the NHS, and that was two and a half years ago, and I haven't had that back yet. I haven’t spoken to anyone.* **P3**  *They [CAMHS] referred me to the autism diagnosis lot when I left them, and it's been sort of sat there since 2018…I don't feel valid [to ask for support] because I haven't got the formal label diagnosis and stuff and a lot of the time when you say I'm awaiting diagnosis it's like ‘no, you need formal diagnosis’ and it's like I can't get that yet - that's a nightmare.* **P6** | *then basically just got told yeah go and do some reading… I'm still at the stage where I'm kind of going through the, the 6 stages of grief with it; well, it's not grief but it's more kind of like ‘yey I have a diagnosis’ and then it's like how much easier could my life have been if I'd been diagnosed earlier? Because within 20 minutes of talking to me the guy was like you are pretty autistic, and he picked up on it really quick and a lot of the kind of struggles I had as a child and that and as a as a young adult it's just been, if I'd known before it would have been so much easier. So, I'm kind of, I'm kind of coming to the end of the anger stage of that.* **P9**  *I then spent the kind of the four years, three, four years since then going backwards and forwards in my head of thinking 'Oh, this makes sense and this explains, a lot of things' and doing loads of research and a lot of reading, and I've got an autistic colleague in my job and we have a lot in common, and going 'oh yeah that makes sense', and other autistic friends, and then, at other times thinking 'oh you're just being completely ridiculous like this is silly of course you're not like you're so different from your friend like you're just making it up', and so I would often get really close to feeling like I wanted to pursue a diagnosis, because I was feeling like it made sense, and then at the last minute I would go 'no you're being silly again' and I wouldn't go ahead with it. And, and I felt like when it started coming back around this time for the last few months, I was like ‘this is ridiculous it's been four years of backwards and forwards, I just, the only way to stop this from going backwards and forwards, is to know’ and, and I thought, for me, the only way to stop that was to actually pursue a formal diagnosis, because although I understand self-diagnosis and I'm certainly not against self-diagnosis, for me it just, it wasn't clear enough, and I was, it didn't stick with me long enough for me to feel like this is a label I could hold on to and identify with, I felt like I needed some external support in working through that.* **P10**  *So, yes diagnosis: useless, in fact damaging. But the group, post diagnosis group: brilliant can't recommend it enough, I think that should be mandatory, you don't just spin people off and leave them with this bit of paper that may or may not be accurate.* **P12** | in *place* there's basically nothing; you get your diagnosis and then they refer you to I think it's *charity* it's a charity, and then they offer you one like meeting to talk about so you can ask questions and stuff, and then that's it. And I don't think that's very good… I think peer support could be a really good thing, is a really good thing, so I think there could be that. Yeah, psychology maybe helping you understand why you do the things that you do, psychology around your strengths and the good things about it. **P21**  *I was always a proud of being different to everybody else, and the identity that I formed for myself, was an identity of being the person who is different. And so, I would actively choose you know not wear the clothes that everybody else was wearing, not because I didn't like them, but because I wanted to be different, and I cultivated that identity of being the odd one out. And then when you get diagnosed, you like, they're like congratulations you're, you think you're really different but actually you're just a cookie cutter version of all these other people who are more, I mean yes, they're very different and autism, but they're basically all the things that you've been holding on to about you that you think are so different those things are all the same as these people, and so being diagnosed stripped me of my identity. And so, initially, it was, I lost identity through being diagnosed, but then you are offered this autistic identity and it's very strange because I've never belonged to a community before.* **P23**  *There isn't really any [support], there's nothing really for adults, particularly, there's only stuff for children and parents support wise and so it's difficult to find any kind of support group that caters to adults who are just needing a bit of someone else to rant to. Not who are, the, the general thing you get for autistic people support wise is a group that will teach you functional skills, and I know how to cook, I know how to clean my house, I know how to *job*, so I have functional skills, what I need is ranting ability. And somewhere to make friends with people who understand… I think it would have been good if, once you finished your diagnosis, they refer you on to a suitable kind of agency or organization or something that you can explore the diagnosis with instead of just being given a diagnosis and then off you go, because that's pretty much what it was; ‘here's your diagnosis, a list of support groups in the area, but most of them aren't really suitable’.* **P20**  *I got diagnosed and then I had like one post diagnostic session…It would have just been nice I don't know just to, Like I don't know I felt like I was diagnosed and then like waved off on my way as, like it'd be nice if I could have just had like a couple more sessions, just like talk it through and just like dunno, process it with somebody.* **P14**  *when I got diagnosed, they offered you like three follow up sessions and I didn't really feel, well I didn't really know what they were for, and I didn't really feel the need for them, because I think I went to one and then I was like, what am I going to do with anymore…and when I started feeling like I need it when everything was getting a bit on top of me…I went to the GP, and I was like can I have some support, because I don't like this whole being autistic thing and, and to their credit, they tried they applied for funding and stuff like that, but they couldn't get anything, and so I just had to plod on.* **P18**  *I guess that was driving me at the time was that you know, I was spending a lot of time researching and thinking about it and I just needed to, sort of I thought find out definitively and then be able to kind of move on, rather than feeling like I was in some sort of limbo. But, unfortunately it wasn't as straightforward as that and the limbo continued for a long time yeah…the idea that you could like reveal this identity is yeah, it's just not, not how it works so yeah I think it's, it's really complicated.* **P15**  *I think it probably changed the most since I've kind of really, kind of accepted being autistic and grown an autistic identity not, not that much sort of happened before that. It was something that, although I knew, I knew I was autistic I didn't really put two and two together. But certainly, in terms of when I started, you start doing any kind of unpacking in terms of identity, that's where things I found the most growth and development if that makes sense.* **P16**  *I felt kind of because it was like 'oh yeah you'll be put on a three-year waiting list', and that was sort of the end of it in a way, I kind of felt like 'Oh, maybe I'm not like that important, like not important, but like not a high priority', you know it kind of made me feel like they just you know put me on a waiting list and then left me to it.* **P24** |
| Having to be the professional | *the most worrying aspect of it is approaching the GP, because I don't know how great the sort of levels of knowledge are about autism, and particularly in women and girls, around sort of a lot of GPs, so it does worry me that if they tried to sort of put me off or try to tell me that I don't need a referral, I'm kind of worried I won't be able to advocate for myself in that situation, despite my giant notebook of evidence.* **P8**  *I think it would have been good to have signposting from one agency to another, so me ringing up the mental health team afterwards I kind of thought ‘well they probably wouldn't know exactly what to do and, and actually it was probably somewhere else’, but they didn't know, so it's like they don't work together, which means that people like me don't get signposted to the right place... But nobody ever said that, so I just had to like Google, I was asking my counsellor and asking all these other people, and it just it's so exhausting.* **P1**  *but he [GP] did just find it a really surprising request, like his response was ‘did you struggle at school, and why hasn't this come to light before and like, why do you need it now?’ kind of thing. And so, I had to say like a few times, ‘like it is really affecting me now’, and he was saying, ‘but if it's not when you were younger’, he was thinking there's no point really whereas my point was, ‘well at the moment I'm so upset that I'm looking at counseling, whereas if this was diagnosed, then there would be no need for counseling because there's nothing really going wrong, that's just the way it is’.* **P2**  *I kept going back to the GP and going it's, something's not right they keep, I keep going and they go ‘yeah, you're, you're depressed have some meds’ and I was like it's not that because I don't feel unhappy but at certain times I can't cope with every day like everyday life, like getting up in the morning's too, too much for me or like my routine's change and I've had a meltdown, and I was like, ‘but I'm not sad about anything I just can't cope right now’, and I kept saying I feel like that it's something else they're not picking up on… I think if the appropriate questions would have been asked you know someone should have picked up on it.* **P4**  *it feels very much like the onus is on you as an individual to make the assessment happen, rather than a medical practitioner saying ‘this is something that we should see’ or we should, you know just anything any kind of update, or any kind of knowledge that they sent you on a referral and you've not heard anything back.* **P3** | *I'd kind of reached crisis point at the end of last year…I cannot wait two years for a diagnosis it's ridiculous on the NHS and my work does private health care, so I sort of negotiated with them to get a diagnosis and to get an assessment um for everything.* **P9**  *my GP said that this was just not her area she didn't know anything about it, why did I think I was autistic, what did I think I would get out of a diagnosis, or an assessment and then she just said, well, you're going to have to go privately, and I said Okay, how do I go about that, and she said just well just Google it. And that was it… I think, with the GP I felt the perhaps I oughtn't have asked, perhaps I'm making a fuss about nothing. And I suppose the reality is I you know she's really busy with COVID and it's not her area, but it kind of rattled me a bit, and that I think kind of slowed me down in terms of seeking out a private diagnosis, because it kind of made me question myself and the research that I'd done.* **P13**  *Yeah, so basically, I went to the doctors and I'd already researched a national autism website like how you should go to the doctors, so I printed off all the information because, like I also know that I'm quite a competent, I can come over as quite a competent person, so I printed off all of the information and took it to the doctors and said ‘I want to have it, an assessment for autism, this is all the reasons why this is the information from National Autistic Society, this is how you should refer me’, so I even told them like what my local area's process was and, and then she said okay I'll refer you on to the next step.* **P11**  *I had approached my GP last year to ask about it and took along, as I had sort of been recommended to me, like lots of research, and information, and notes that I'd made, and was referred to the local talking therapies place - the GP clearly didn't know what to do and just went 'okay well these people might be able to help you with a diagnosis, I don't know' - and they offered me six sessions of CBT for anxiety, and I was like ‘cool, no thanks’…I knew that it was really unlikely that I'd even be successful in getting a GP to refer me for an assessment, and let alone, as I say, the long waiting list and things, so I thought well going private is kind of the only option.* **P10** | I was under the mental health team because I had quite bad depression and we somehow ended up having a conversation about autism and then she, the psychiatrist, said she'd been thinking that anyway, but she didn't want to mention it to me in case I guess in case I freaked out, I don't know, so when I said it to her that sort of started the conversation… but then she found out that she couldn't do it, it had to be my GP, so she thought my GP was doing it, and then my GP thought the psychiatrist was doing it and actually nobody did it, so that stuck a whole year on to the thing, on to the whole process which was really frustratinsg. So, when they figured out who was doing it, I think my GP did it in the end, it took about a year. **P21**  *I was in an inpatient psychiatric Ward for 14 months for people with borderline personality disorder, and I came out in *year* and, and I remember, obviously it took a few years to kind of find myself and work out who I was again, and once I was out the mental health services mostly, I remember kind of thinking about my behavior when I was in the hospital and the behaviour of the other women in the hospital and how very different my behavior was from theirs, and so I thought that it was worth pursuing a diagnosis. And I know that my, that autism can come, well sometimes BPD sometimes maybe be misdiagnosed as autism, especially in females because it's the label that you get put on you and so I thought I would pursue it.* **P20**  *I went to my GP, and I didn't really know what say, but I just knew something wasn't OK, and something needed to stop, and he, he started off saying, because I go to work every day, that means everything's fine and to me that wasn't, that wasn't really true, and I think maybe because, because of my autism, I found it really hard to not go to work every day like that was my, that was what I did, and if that meant I needed to hurt myself to, to show, everyone that I was doing that, that, that would be okay…I went to my next appointment with my psychiatrist, and I just said to her, the first thing I said to her was, she said ‘how are you’ and I said ‘I think I've got autism’ and then she just said, ‘I agree’, and there was like this big like a bit of a relief and maybe she'd been thinking about it as well and so yeah, that was, I wasn't diagnosed at that moment but that's when, that's when autism started entering my head.* **P22**  *But somebody to do I guess to do some of the therapy work that I've done since then in kind of exploring your beliefs about yourself and why you hold those beliefs and what you can do what you know whether they're really true or not.* **P18**  *I decided to use a private psychology service, partly because I'd had quite negative experiences with healthcare professionals over the years when it comes to, I guess what I now know, is a misdiagnosis of what were seen as mental health conditions, and that had all been quite awful so I, I decided to go private and I did a lot of research, about the person that I eventually ended up seeing.* **P15**  *I can do that a lot better than I used to. I'm sure, like the therapist would want to be like oh yeah that's because of me, and you know or the psychiatrist, but I think honestly, I've done a lot of the work myself.* **P17**  *When I interviewed for a job, I didn't tell them because I was worried that it would impact on that, and I kind of wish I had told them… I think it might have helped me with better support, but at the same time, I was just so worried about like being turned down for it because I went for a volunteer job once and put on that I was on the autistic spectrum and I got turned down, and I do think it was because of that. So, sometimes when I'm comfortable with people I tell them that I've got the diagnosis, and then like in job interviews I won't tell them until it's like needed, they need to know.* **P24** |
| No one saw me | *I guess a lot of why I went unsupported is to do with the fact that I was quite quiet, I didn't misbehave, and I was quite academically able, so I was getting good grades, and that seemed to be the thing that mattered. And then, when my sort of my quietness became more of a problem it tended to be perceived as like a difficult behavior that I was sort of willfully choosing to do, and not something that needed to be investigated and supported, and I feel like there were, there were opportunities for my school and my parents to have noticed that you know I was so quiet, and so kind of ostracised at school, and so kind of unhappy. But I just kind of tended to get criticized for not making enough effort to fit in again, as if this was like a deliberate choice of mine.* **P8**  *I was diagnosed with borderline personality disorder or BPD. And when I told my counsellor, who is in fact female and autistic, she said, ‘I don't think you are, I've thought since we met that you're autistic’.* **P1**  *I was considered very weird different difficult and, and my mum thought that I was being, someone has done some magic, black magic to me…there was no black magic, there was nothing, it was just a plain autistic child and misunderstood and that's it, but I mean it was hell and I still, I feel, in my mind like a lack of self-esteem completely, because I mean for all my childhood I was told that I wasn't acting, that wasn't my fault, or whatever I could do it wasn't me it was someone doing this for me.* **P5**  *I think they have a really like one dimensional view of it, like Sheldon from big bang theory, so they would straightaway be like ‘no, like we have good conversations, like you look at me in the eye, you can't, can't possibly be that, you're just trying to do something different’.* **P2**  *I think it was definitely not picked up because I was a woman… I'm very high functioning, I think people think that I'm like making an excuse or I'm like making it up… I do feel a bit let down sort of for my whole adult life I'd been told that I was mentally ill and actually no that wasn't the case I was perfectly healthy.* **P4**  *I think a diagnosis helps one: boost awareness, and two: help the actual person who's being diagnosed yeah if that makes sense.* **P6** | *I think before it was the everyone seems to have that kind of image of like the typical autistic person is like Sherlock Holmes or Sheldon Cooper, and if you don't identify with that, you're probably not autistic…it's not actually, as you know, black and white as the media might make it out to be.* **P9**  *because my idea of autism was Rain Man, it was the little boy with trains and maths and so on, and I think probably as most people's idea at the moment.* **P13**  *I mean I guess the things that made me think 'well you know you can't possibly be autistic' would be things like, like it was all based on stereotypes of autism, I think, or kind of the sort of most yeah stereotyped traits; so, things like I have friends, I was like ‘well there you go’, completely neglecting the fact that my autistic friend who led me to think this is my friend, therefore, they have friends, like and I can make eye contact…those would be the things, and I think those mainly, as I say, grew out of comparison with one particular individual and comparison with the sort of stereotypes of like young boys who like trains and can't make eye contact.* **P10**  *For 33 yrs I felt I’d failed doing my best towards my degree. Unable to repeatedly ask for and obtain practical help at university (*degree*) or cope with the daily tube journey, I built myself a bench, got a book (pre-internet) and worked it out myself, at home. There were no accommodations made then and in spite of being told ‘this is not a correspondence course’ and being marked well down for ‘absenteeism’ I passed. My work was considered good. I now see my disempowerment in a new and positive light though I wonder how life -and how I saw myself- might have been different had those accommodations been made?* **P12**  *You know all this inclusivity and making things accessible, they won't work unless you've got people who are doing the including and helping are autistic, otherwise it doesn't work 99% of the time. Yeah, it just doesn't work. You need people who are autistic actually in there you know doing the spade work or it doesn't work.* **P12** | *I think people still think that it's not found in women and girls, and I think that sometimes makes me not feel confident in identifying it in different situations.* **P21**  *I think the majority of people are still dismissive and will just say well ‘okay, well it's over diagnosed, these days, anyone can get any diagnosis’ and it's often also dismissed that because I had a private diagnosis thing you know, like just paid for a piece of paper that I wanted, or like ‘you're not, you're not properly autistic, you don't speak for all autistics’, but you know, yeah or ‘well it's just it's just you know navel gazing just get on with it’, whatever. But yes, some people, I mean you know some, some people are understanding so, but the vast majority, I would say are not… I think, from, from other people is just general ignorance. My, I have a fairly, relative, distant ish family member who is a clinical psychologist who diagnoses autism, and it's, it's quite shocking and, but, but they are adamant that you know I'm not autistic.* **P19**  *because people have a very certain view of what an autistic person looks like; it's usually a male, a child, because the children can't grow up to be adults. And so, I've had people say to me ‘you're not autistic’ because they assume because I don't look autistic, I'm not autistic, and just completely deny my diagnosis.* **P20**  *he used to say to me things like ‘do you think you've got ADHD’ or, so there still wasn't specifically, autism wasn't mentioned, but there was still people suspecting that there was something, something going on that wasn't quite working out.* **P22**  *straight after being diagnosed, I felt really kind of like lost for a bit and like quite low because, if I had found this out like way, way, because I was got diagnosed quite late like I was in my late 20s, like if I had known this much earlier than like life could have been, could have saved so much struggle like earlier on in life, so I felt a bit of like I guess grief for like not knowing and like my life that I lived.* **P14**  *I vaguely mentioned it [thought of being autistic] to my mum who was like 'don't be so ridiculous' and so, then I shut up about it again.* **P18**  *the doubts; the fact that no one had ever suggested it might be a possibility, even though I'd had ongoing contact with you know, mental health professionals, GPS etc. over the years.* **P15**  *I think a lot of people involved had a lot of internalized ableism and didn't want to seem that someone who was relatively intelligent was disabled… their assumption of what an autistic person is projected onto me from one person they saw on a TV show or one person they know from a family friend or one person they studied in a class once or the one person they've met down the shop, rather than actually understanding the true spikey profile and difference of needs that we all have and how we differently present in different contexts.* **P16**  *I was in mental health services and going from one diagnosis to the next and I didn't really even considered that I might be autistic…I'd been with them for like 18 months and then sort of on the way out, they were like oh yeah, by the way, we think you might be autistic.* **P17**  *when like I've told people that I'm autistic they always go ‘oh, but you are so well behaved’ and you know, ‘but you know you're so like academically smart’ and stuff because there's still quite like a stigma of it being you know, like you're a naughty child or something like that, and there's still like, you know I've had one person say to me ‘but you're a girl’, which was kind of like a huge like sort of like ‘oh wow, you still think that’ it's just like, which is kind of sad, in a way.* **P24**  *At the end, she told me she said, like I'm certain that you're autistic and then she told me that her daughter is and she's like you, like the way you are reminds me so much of her, so I think that's why I felt understood by her.* **P14** |
